# The Effect of Habitual Fat Intake, IL6 Polymorphism, and Different Diet Strategies on Inflammation in Postmenopausal Women with Central Obesity

**DOI:** 10.3390/nu11071557

**Published:** 2019-07-10

**Authors:** Agata Chmurzynska, Agata Muzsik, Patrycja Krzyżanowska-Jankowska, Jarosław Walkowiak, Joanna Bajerska

**Affiliations:** 1 Institute of Human Nutrition and Dietetics, Faculty of Food Sciences and Nutrition, Poznan University of Life Sciences, Wojska Polskiego 31, 60-624 Poznań, Poland; 2 First Subdepartment of Pediatrics, Department of Pediatric Gastroenterology and Metabolism, Poznan University of Medical Sciences, Szpitalna 27/33, 60-572 Poznan, Poland

**Keywords:** TNFα, IL6, single nucleotide polymorphism, postmenopausal women, dietary fat, dietary intervention

## Abstract

The hypothesis that habitual fat intake, the *IL6* genotype, the Mediterranean diet or the central European diet for 16 weeks affect biomarkers of inflammation in centrally obese postmenopausal women, was tested in a randomized controlled trial. Dietary intake was assessed using a three-day food diary. Lipid parameters were measured using a Beckman Coulter AU analyzer. Transcription of *TNF* and *IL6* genes was analyzed in peripheral blood mononuclear cells using real-time PCR. Concentrations of tumor necrosis factor alpha (TNFα) and interleukin 6 (IL6) were measured with ELISA. rs1800795 polymorphism of *IL6* was analyzed using hydrolyzing probes. Higher energy intake from fat was associated with higher IL6 levels (*p* < 0.05). Significantly (*p* < 0.01) lower total cholesterol (T-C) and low-density lipoprotein cholesterol (LDL-C) concentrations were observed in the GG *IL6* rs1800795 genotype group. Both diets significantly (*p* < 0.001) decreased TNFα concentrations. Neither *IL6* gene transcription levels nor blood IL6 concentrations were affected by them. Our findings confirm that habitual fat intake may affect inflammation. The rs1800795 *IL6* polymorphism alone did not significantly affect body weight or body composition in aimed group, but C-allele carriers had higher levels of T-C and LDL-C. This polymorphism did not affect inflammation. Both diets may lead to a decrease in TNFα concentration.

## 1. Introduction

Obesity is associated with systemic, low-grade inflammation, which may play an important role in the pathogenesis of obesity-related insulin resistance, dyslipidemia, and the atherosclerotic process [1,2]. White adipose tissue (WAT) acts as lipid storage, but also as an endocrine organ that releases signaling molecules called adipokines; these include leptin, adiponectin, and the main cytokines responsible for chronic inflammation—namely tumor necrosis factor alpha (TNFα) and interleukin 6 (IL6) [3,4]. TNFα is primarily secreted from monocytes and macrophages, and plays a major role in inflammation, immune system development, apoptosis, and lipid metabolism [3,5]. IL6 is involved in inflammation and infection responses, but a role of IL6 in the control of lipid metabolism has also been confirmed in several observations [6]. The *IL6* gene is expressed in many tissues, including adipose tissue, skeletal muscles, and the hypothalamus—all of which are involved in the regulation of energy balance [7]. Higher circulating levels of IL6 have been associated with obesity and visceral adiposity [8,9,10]. Body weight—and especially centrally localized adipose tissue—as well as fat intake are among the modifiable factors that affect inflammatory biomarkers [11]. There are studies showing an increase in pro-inflammatory serum markers, especially IL6 and TNFα, after menopause and suggested that in addition to age these changes have been attributed to estrogen decrease [9,10]. This is associated with an increased incidence of cardiovascular diseases among the targeted population. Therefore it is important to develop beneficial dietary treatment for postmenopausal women. There are several polymorphisms of the *IL6* gene, with rs1800795 being the most frequently studied. This single nucleotide polymorphism (SNP) is located in the promoter region, 174 bp upstream of the transcription start site, and it affects gene activity. The C allele is associated with a lower expression level [11]. Although the results of studies on how rs1800795 affects inflammation, body weight, and adiposity do conflict, it has been suggested that the C allele is associated with higher adiposity and lower energy expenditure [12,13]. Studies of dietary therapies that may reduce the inflammation associated with central obesity are limited. The effectiveness of the Mediterranean diet (MED) and its main components (mainly monounsaturated fatty acids, MUFAs) in reducing inflammation, in subjects with metabolic syndrome, has been demonstrated [2,14]. Whether and how the alternative dietary regimen (moderate in carbohydrates, and dietary fiber) composed into the central European diet (CED) mitigates the inflammatory state in postmenopausal women with central obesity remains unknown. The aims of this study were thus: (1) to analyze the effect of habitual fat intake on inflammation biomarkers, including transcription of *TNF* and *IL6* genes, and blood concentrations of IL6 and TNFα; (2) to analyze the effect of the *IL6* polymorphism on anthropometric parameters and biomarkers of inflammation of targeted population before dietary intervention; and (3) to assess how dietary interventions differing in fat, carbohydrate, MUFAs and dietary fiber content may affect these parameters in targeted population. CRP was not analyzed as its concentrations correlate with IL6 in a general population, and in postmenopausal women.

## 2. Materials and Methods

### 2.1. Study Design and Subjects

The study design and participants have been described in detail elsewhere [15]. Shortly, this study was conducted as a randomized controlled trial (DRKS00012958; https://www.drks.de/drks_web/) with a parallel arm design and 1:1 allocation. The study methods and reporting were conducted in accordance with the CONSORT 2010 guidelines. The participants were randomized to the CED group or the MED group for 16 weeks. The details composition of the diets are presented in Table 1.

**Figure 1:**
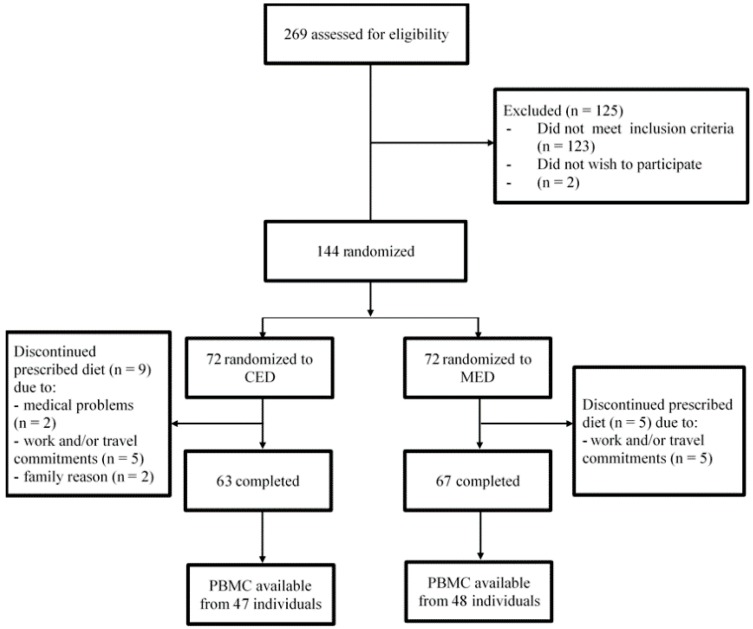
Flow diagram.

Shortly, both studied diets were hypocaloric with an energy deficit aiming at a minimum weight loss of 0.7 kg per week. The MED had moderate levels of fat (providing 37% of total energy) derived mostly (20% of energy) from MUFAs, where the main source of fat was virgin olive oil and nuts. The CED had lower than 10 percentage points of energy from fat (27% of total energy), and moderate levels of carbohydrates (55% of total energy) derived mainly from high-fiber locally available and traditional food items (e.g., rye bread, oatmeal, barley, apples, plums, beetroots, etc.). Therefore, the CED was characterized by higher dietary fiber content than the MED, mostly in terms of soluble dietary fiber [15]. Moreover, the study diets were similar in protein, saturated fatty acid (SFA) and polyunsaturated fatty acid (PUFA) contents with 18% energy from protein, 8% from SFA and 9% from PUFA, respectively. Added salt and refined fats, as well as sugar, were excluded from both diets. A fourteen-day cyclic dietary plan were formulated. During the entire dietary intervention participants picked up packaged main meals (covering ~35% daily energy requirements) prepared according to the dietary plan. Others meals were prepared by the study participants themselves. All the subjects were advised to maintain their usual level of physical activity (PA) and keep other lifestyle factors unchanged. PA was assessed with the short form of the International Physical Activity Questionnaire (IPAQ-SF). All study participants provided their written informed consent, and the local ethics committee at Poznan University of Medical Sciences (number 603/14) approved the study protocol. Women who experienced a natural menopause were invited to participate in this study. Postmenopausal state was defined as at least 1 year since last menstrual period plus a serum follicle-stimulating hormone concentration of 30 IU/L or higher at screening. Additionally, the inclusion criteria were central obesity (waist circumference ≥80 cm) plus at least one other of the International Diabetes Federation criteria for metabolic syndrome [16]. Women who smoked, with type 2 diabetes; monogenic dyslipidemia; with a history of cardiovascular disease; using of hypoglycemic, hypolipidemic, anti-inflammatory, or weight loss agents, as well as any drug known to influence liver function; with endocrine disorders or on hormonal replacement therapy, were not eligible. Upon entry to the study, the postmenopausal women were on average 60.5 year old, while their average age at menopause was 51.0 years. Altogether, 269 individuals were originally contacted and screened, and 144 were randomized as described earlier [15]. In this present sub-study of the CED-MED trial, we included a total of 95 subjects, aged 60 (0.5); *n* = 47 in the CED and *n* = 48 in the MED out of the 130 subjects who completed the CED-MED study. We excluded 35 subjects because we had no access to peripheral blood mononuclear cells (PBMC) samples (Figure 1).

### 2.2. Dietary Assessment

Nutrient intake was assessed using a three-day food diary in which participants were clearly instructed to record information on nonconsecutive days (two weekdays and one weekend day) on their food and beverage intake, using household measures. The average intake of macronutrients and micronutrients at baseline and during the dietary intervention was calculated using the Dietetyk software (Jumar, Poznań, Poland).

### 2.3. Anthropometry

Height was measured to the nearest 0.1 cm (RadWag, Radom, Poland). Body weight was measured, using a calibrated scale included in the Bod Pod technology, to the nearest 0.1 kg. Waist circumference was measured at the midpoint between the lowest rib and the top of the iliac crest using nonelastic tape. The BodPod was used in line with the manufacturer’s instructions to measure body volume. All subjects wore tightly fitting bathing suits and swim caps.

### 2.4. Analysis of Transcription Levels in PBMCs

Immediately after blood collection, PBMCs were isolated using density gradient centrifugation with Lymphoprep (Stemcell Technologies, Vancouver, BC, Canada). Whole blood was diluted with sterile PBS, layered over Lymphoprep, and centrifuged at 400× *g* for 20 min. The PBMC buffy coats were collected, washed twice with PBS, and immediately proceeded to an RNA isolation procedure. Total RNA was extracted from PBMCs using Tripure Isolation Reagent (Roche, Basel, Switzerland), according to the manufacturer’s protocol. Approximately 1 μg RNA was taken for cDNA synthesis. Samples of RNA were incubated with a set of random hexamers (Roche, 0.25 μg/μL) and oligodT(_15_) (Roche, 0.25 μg/μL) at 70 °C for 10 min. A mixture of dNTP (5.0 mM, Roche), 1 U reverse transcriptase AMV (EUR_X_, Poland), and 20 U Protector RNase Inhibitor (Roche) was added. After two hours of incubation at 37 °C, the AMV enzyme was inactivated at 94 °C for 5 min. The cDNA was then diluted 3 × and stored at −20 °C. The real-time PCR reactions were performed on a Light Cycler 480 (Roche), based on the Universal Probe Library detection system (Roche, Table 2).

Each 10 μL reaction mixture consisted of 1 μg cDNA, 5 μL LightCycler 480 Probe Master kit (Roche), 0.2 μL of the respective of the Universal Probe Library (UPL), and 0.3 μM forward and reverse primers. The real-time PCR was performed according to the manufacturer’s protocol. The relative quantification of the mRNA level was performed in duplicate based on a Second Derivative Maximum Method (Roche). Standard curves were designed as tenfold dilutions of the appropriate PCR product in the range of 10–0.0001 aM. The abundance of *IL6* and *TNF* gene transcripts was then normalized to a geometric mean of two reference genes using glyceraldehyde-3-phosphate dehydrogenase (*GAPDH*), and β-actin (*ACTB*) [17]. 

### 2.5. Measurement of Blood Biomarkers

TNFα and IL6 concentrations were estimated in serum using an enzyme-linked immunosorbent assay method (TNF-alpha ELISA, DRG Instruments GmbH, Marburg, Germany; high sensitivity IL6 ELISA, R&D Systems, Minneapolis, MN, USA), according to the manufacturer’s directions. Concentrations of total cholesterol, high-density lipoprotein cholesterol (HDL-C), triglycerides (TG), and glucose in plasma were measured using a Beckman Coulter AU analyzer. Low-density lipoprotein cholesterol LDL-C concentrations were calculated using the Friedewald formula [18]. rs1800795 of the *IL6* gene was selected for analysis. The blood was collected into tubes containing EDTA and DNA was isolated from fresh blood using a NucleoSpin Blood kit (Macherey–Nagel, Germany). Genotyping was performed with the use of hybridizing probes designed and synthesized by TibMolBiol, Germany (Table 1). PCR was run using the Probe Master kit (Roche) at a primer concentration of 0.5 µM and a probe concentration of 0.15 µM. The reaction was performed in 96-well format with a total reaction volume of 10 µL, using 20 ng of genomic DNA. The cycling profile was 95 °C for 10 min, followed by 45 cycles at 95 °C for 5 s, 57 °C for 10 s, and 72 °C for 15 s. Genotypes were analyzed using a melting curve method.

### 2.6. Statistical Analysis

Normality of the data was tested using the Shapiro–Wilk test with α = 0.05. For data with non-normal distribution (gene expression data), the differences between the parameters before and after the DI were assessed using the Wilcoxon test. Between-group differences were tested using the Mann–Whitney test. Data with normal distribution were analyzed using Student’s *t* test. Adjustments for PA and BMI were made by linear regression. To analyze the effects of nutrient intake at baseline on the parameters, the study group was divided into subgroups based on median values of energy derived from fat, protein, or carbohydrates, as well as total intake of fat, saturated fatty acids (SFAs), MUFAs, and PUFAs. *p* < 0.05 was taken to be statistically significant. Data were analyzed using Statistica software (StatSoft, Tulsa, OK, USA).

## 3. Results

The detailed results on how the DI affected anthropometric parameters, as well as glucose and lipid metabolism, have been described elsewhere [15]. Here we focus on the inflammation biomarkers. We first analyzed how the usual intake of different types of fat affected baseline values of inflammation biomarkers in a group of postmenopausal women with central obesity. Transcription levels of *IL6* and *TNF* in PBMCs before the DI were not affected by baseline energy intake from fat, total fat intake, PUFA, or MUFA intake. However, lower intake of SFA was associated with an over 500 times lower *TNF* transcription (*p* < 0.05). Specifically, the median values of the *TNF* relative transcription levels were 0.000576 and 0.000001, in the low and high SFA groups, respectively. The amount of baseline energy intake from fat affected the initial IL6 concentrations (*p* < 0.05). Specifically, the high energy intake from fat was associated with higher IL6 levels (see Table 2). Moreover, high energy intake from protein resulted in higher blood TNFα concentrations (*p* < 0.01). The intake of SFA, MUFA or PUFA did not affect IL6 or TNFα levels (Table 3).

We also examined the associations between the *IL6* polymorphism and baseline anthropometric and lipid parameters or inflammation biomarkers. The genotype frequencies for rs1800795 were 0.25 (GG), 0.56 (GC), and 0.19 (CC), and the allele frequencies were 0.53 (G) and 0.47 (C). The *IL6* genotype groups differed in transcription levels at baseline, but only when SFA intake was considered (*p* < 0.05). Specifically, when SFA intake was high, the transcription level was over three times higher in the C-allele carriers group than in the GG genotype group. There was no effect of *IL6* polymorphism on the anthropometric parameters (Table 4). Lower total cholesterol and LDL-C concentrations were observed in the GG genotype group (*p* < 0.01 for both associations; Table 4). These associations were also observed after adjustments for PA and BMI. The *IL6* genotype was not associated with concentrations of any of the measured inflammation biomarkers.

The last step of the study was to determine how the dietary intervention affected inflammation biomarkers and whether the effect was dependent on the type of diet (CED or MED). 

**Figure 2:**
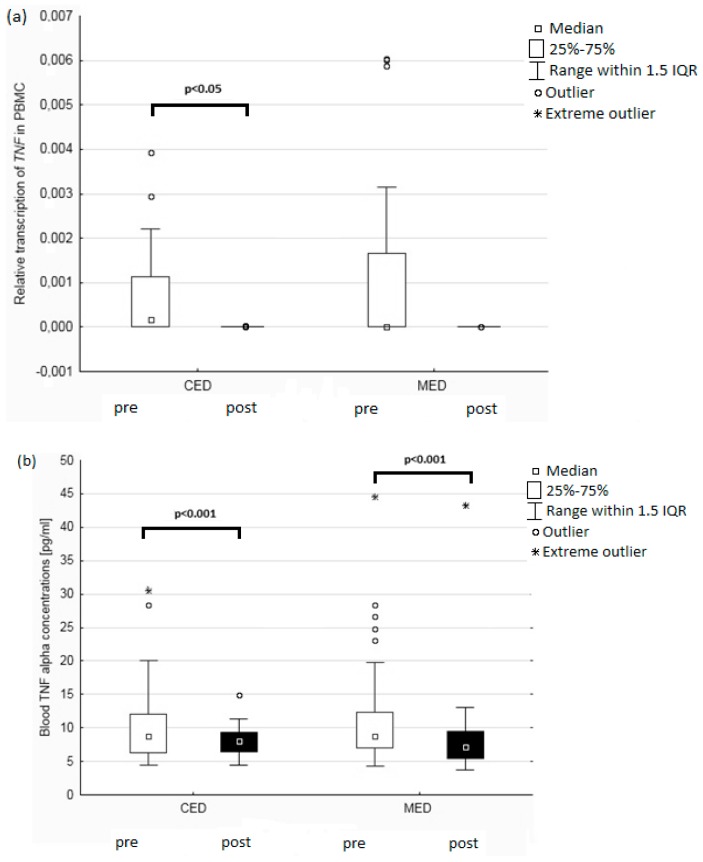
The mRNA levels of TNF expression in PBMCs (**a**) and blood TNFα concentrations (**b**) pre and post the intervention among 95 postmenopausal women. Boxplots shows median values and interquartile ranges. Circles represent outliers that extend more than 1.5 box-lengths from the edge of the box, and stars represent extreme outliers which extend more than three box-lengths. The differences between the parameters before and after the dietary intervention were assessed using the Wilcoxon test. PBMC: peripheral blood mononuclear cells; CED: Central European diet; MED: Mediterranean diet; IQR: interquartile range.

We measured *TNF* and *IL6* genes expression in PBMCs, and the transcription levels of the *TNF* gene decreased in both groups after the dietary intervention, but it was statistically significant only in the CED group (*p* < 0.05; Figure 2a) or when both groups were analyzed together (*p* < 0.001). Both types of dietary intervention also led to a decrease in blood TNFα concentration—in the CED by 9% and in the MED group by 17.5% (Figure 2b). However, the type of diet used had no significant effect on the results. TNFα concentrations before and after the dietary intervention were correlated: 0.58 and 0.60 in the CED and the MED group, respectively (*p* < 0.001). 

**Figure 3:**
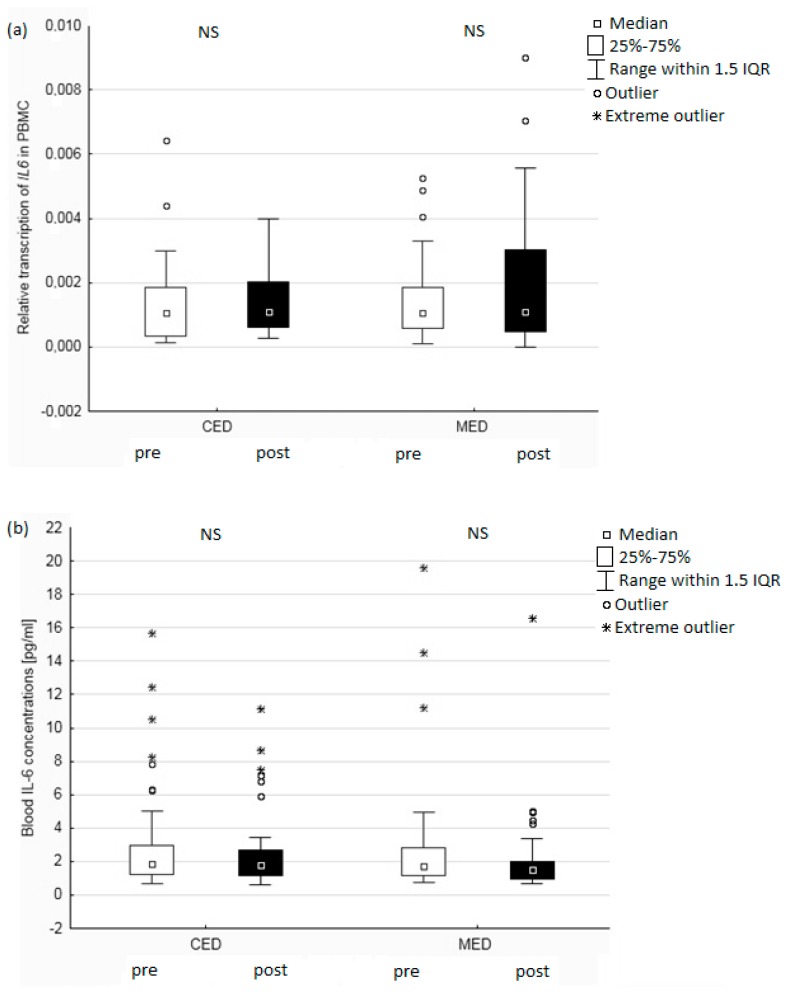
The mRNA levels of *IL6* expression in PBMCs (**a**) and blood IL6 concentrations (**b**) pre and post intervention among 95 postmenopausal women. Boxplots show median values and interquartile ranges. Circles represent outliers which extend more than 1.5 box-lengths from the edge of the box and stars represent extreme outliers which extend more than three box-lengths. The differences between the parameters before and after the dietary intervention were assessed using the Wilcoxon test. PBMC: peripheral blood mononuclear cells; CED: Central European diet; MED: Mediterranean diet; IQR: interquartile range; NS: non-significant.

*IL6* gene transcription levels were not significantly affected by any of the diets (Figure 3a). We also did not observe any significant decrease in blood IL6 concentrations, in either the CED or the MED group (Figure 3b). The only correlation between gene expression and blood cytokine concentrations was a reverse correlation between *TNF* transcription and TNFα concentrations before the intervention (*r* = −0.45, *p* < 0.05).

## 4. Discussion

We hypothesized that habitual dietary fat intake, the IL6 genotype, and MED or the CED for 16 weeks would affect selected biomarkers of inflammation (TNFα and IL6) in postmenopausal women with central obesity; the obtained results partly confirm this hypothesis. We observed that, before the dietary intervention, lower habitual intake of SFA was associated with lower TNF transcription, but not with blood TNFα concentrations. Although no associations between fat or fatty acid (FA) intake and IL6 transcription in PBMCs were detected, blood IL6 concentrations were higher in postmenopausal women whose percentage energy from fat was higher. Our results are convergent with a review presented by Telle-Hansen et al. in 2017, where it was stated that most dietary interventions showed either no or minor effects of dietary fat intake on inflammatory markers in overweight and obese subjects [19]. We also observed that a higher percentage of energy consumed as protein was associated with higher TNFα plasma concentrations. This relation may be attributed to higher intake of end products of processing protein-rich foods, which are associated with oxidative stress and inflammation [20]. TNF and IL6 are target genes of PPARγ, which is one of the main regulators of lipid metabolism, and may be activated by FA [21]. This might explain the association observed in our study between habitual SFA intake and TNF transcription. On the other hand, these cytokines are secreted not only by monocytes (where gene expression was measured in this study); they are also produced by macrophages that infiltrate adipose tissue [22].

Moreover, the dietary intervention led to decreased *TNF* transcription in PBMCs and decreased blood TNFα concentrations; the effects of both diets were similar, suggesting that observed result was mainly due to weight loss. Bianchi in his 2018 review concluded that in obese and overweight subjects weight loss, induced both by energy-restricted diet or surgery, is a determinant factor for reducing the level of pro-inflammatory markers [23]. Also, Smidowicz & Regula in 2015 as well as Imayama et al. in 2012, suggested that the main trigger for the improvement of inflammatory biomarkers, even among postmenopausal women is weight reduction, independent of the type of an intervention [24,25].

We did not observe any effect of either analyzed diet on *IL6* transcription or blood IL6 concentrations, which indicated that this inflammatory pathway remained activated even after weight loss. It has previously been shown that the Nordic diet downregulates the expression of genes involved in inflammation in subcutaneous adipose tissue in subjects with MS, but not *TNF* and *IL6* [26], while a diet rich in SFA has been shown to increase the expression of proinflammatory genes in the adipose tissue of people at risk of MS; again *IL6* was not among these genes [27]. Blomqvist et al. performed a study on postmenopausal women with obesity; this was similar to ours, but they used Paleolithic-type and prudent control diets [28]. There was no caloric restriction, and the study diet was based on lean meat, fish, eggs, vegetables, fruits, berries, nuts, avocado, and oils. Partly similarly to our results, the intervention resulted in a relatively quick body mass reduction, but significant reductions in blood TNFα and IL6 concentrations were observed after 24 month of the trial. As IL6 concentrations depend on the time of dieting [21], it seems the duration of our study was most likely too short to detect any changes. However, Richard et al. observed a 20% reduction in IL6 concentrations after 20 weeks of MED which was aimed at decreasing body weight in men, diagnosed with MS [14]. On the other hand, in a study conducted by Mališová et al., 20 weeks of very low calorie diets resulted in no changes in IL6 concentrations in obese perimenopausal women [29].

Since the rs1800795 polymorphism regulates *IL6* gene expression, we hypothesized that it may significantly affect blood IL6 concentrations, but the differences in the transcription levels in different genotype groups were not statistically significant. The blood IL6 concentration was also unaffected by the *IL6* genotype, which is in agreement with previous studies that suggest that *IL6* expression regulation does not only depend on this polymorphism [30]. Similarly, in the EPIC-Potsdam study, IL6 concentrations were elevated in obese subjects, but the levels of this cytokine did not differ in the *IL6* genotype group [31]. A meta-analysis has shown that the C allele of rs1800795 is associated with increased adiposity [32]. In the PREDIMED study, CC subjects had higher body weight than people with GG or GC genotype [13]. Possibly due to the sample size being too small, no genotype effect on body weight or adiposity was observed in our study. Moreover, all women in the present study had central obesity and interindividual variability was thus relatively low. It may be anticipated that differences between the *IL6* genotype groups could be observed when comparisons are made between lean and obese subjects. Moreover, Joffe et al. showed that, with increasing PUFA, docosahexaenoic acid and eicosapentaenoic acid intake, BMI lowered in subjects with the C allele, also with increasing MUFA intake, TG concentrations decreased in the C allele carriers [8]. The effect of the MED on weight loss was also *IL6* genotype-dependent [13]. This suggests that gene × diet interactions may play a significant role in the effect of rs1800795 on body weight. In our study, the *IL6* genotype was associated with T-C and LDL-C concentrations, while GG subjects had lower values of these parameters. Surprisingly, Henningsson et al. observed associations between the *IL6* genotype and lipid profile, but in the opposite direction—women with the CC genotype had lower levels of cholesterol and LDL-C [30]. Altogether, this may suggest that higher values of T-C, LDL-C or inflammation biomarkers are mostly due to obesity, but not IL6 polymorphism, and that fat intake significantly modifies the gene effect on lipid metabolism.

Our study has several limitations, which include lack of analysis of how gene × diet interactions might have affected the effectiveness of the intervention. The study was based on two different diets, and the total number of participants was too small to this type of analysis. Moreover, although adipose tissue is an important source of TNFα and IL6, especially in obese patients, we did not have access to samples of adipose tissue for gene expression analysis. However, this approach has been successfully applied earlier by other groups [33,34].

## 5. Conclusions

Blood TNFα and IL6 concentrations are not significantly affected by the transcription levels of their respective genes in PMBCs. Higher energy intake from total fat or protein may be associated with elevated concentrations of circulating plasma IL6 and TNFα, respectively. The rs1800795 *IL6* polymorphism alone does not affect body weight or composition in targeted population. Most likely, this is due to very small gene effects that cannot be detected in a relatively small group with low inter-individual variability. The C-allele carriers have higher levels of T-C and LDL-C. This polymorphism does not significantly affect blood inflammation biomarkers. Both diets, regardless of macronutrient contents lead to reducing inflammation biomarkers in postmenopausal women with central obesity.

## Figures and Tables

**Table 1:** The composition of the both compared diets.

Type of Diet	CED	MED
Carbohydrates % E	55	45
Protein % E	18	18
Fat % E	27	37
SFA % E	8	8
MUFA % E	10	20
PUFA % E	9	9

% E: percentage energy from macronutrients.

**Table 2:** Probes and primers used for gene expression and genotyping analysis.

Gene	Names of Oligonucleotides *	Primers and Probes
Gene expression		
*IL6*	#40	5′ gatgagtacaaaagtcctgatcca 5′ ctgcagccactggttctgt
*TNF*	#29	5′ cagcctcttctccttcctgat 5′ gccagagggctgattagaga
*GAPDH*	#60	5′ agccacatcgctcagacac 5′ gcccaatacgaccaaatcc
*ACTB*	#64	5′ ccaaccgcgagaagatga 5′ ccagaggcgtacagggatag
SNP analysis		
*IL6* rs1800795	IL-F	5′ ttactctttgtcaagacatgcca
	IL-R	5′ atgagcctcagacatctccag
	IL Anchor	5′ ctaagctgcacttttccccctagt
	IL Sensor	5′ gtgtcttgcgatgctaaagga

* For gene expression analysis, the name of each oligonucleotide refers to the number of the Universal Probe Library (UPL) used.

**Table 3:** Effect of initial nutrient intake on baseline blood cytokine concentrations.

Intake	Median Intake	TNFα [pg/mL]		*p*-Value	IL6 [pg/mL]	*p*-Value
Energy from fat [%kcal]	36%	low	9.73 ± 6.03	0.184	2.24 ± 2.83	0.040
		high	11.53 ± 7.11		3.64 ± 3.70	
Energy from proteins [%kcal]	17%	low	8.71 ± 2.52	0.005	2.39 ± 2.37	0.126
		high	12.46 ± 8.59		3.44 ± 4.03	
Energy from carbohydrates [%kcal]	46%	low	11.39 ± 7.12	0.254	3.40 ± 3.50	0.167
		high	9.83 ± 6.02		2.45 ± 3.14	
Total fat [g/day]	75	low	9.87 ± 6.54	0.286	2.92 ± 3.73	0.996
		high	11.32 ± 6.65		2.92 ± 2.94	
SFA [g/day]	27	low	10.63 ± 7.57	0.967	3.14 ± 3.69	0.529
		high	10.58 ± 5.56		2.70 ± 2.98	
MUFA [g/day]	29	low	10.02 ± 6.48	0.396	2.90 ± 3.74	0.949
		high	11.18 ± 6.73		2.94 ± 2.94	
PUFA [g/day]	11	low	10.59 ± 6.71	0.976	2.85 ± 3.69	0.834
		high	10.63 ± 6.54		3.00 ± 3.09	

Results are shown as group means with their standard deviations. TNFα: tumor necrosis factor alpha; IL6: interleukin 6; SFA: saturated fatty acids; MUFA: monounsaturated fatty acids; PUFA: polyunsaturated fatty acids. Low intake here means intake below a median value, with high intake referring to intake above that median value.

**Table 4:** Biomarker levels by *IL6* variants at baseline.

Parameter	rs1800795 Genotype Groups		*p*-Value
	GG	GC and CC	
Body weight [kg]	82.6 ± 10.6	83.6 ± 12.0	0.395
Waist circumference [m]	1.03 ± 0.07	1.03 ± 0.08	0.888
% fat mass	48.6 ± 4.8	48.3 ± 4.8	0.525
BMI	32.8 ± 3.8	32.9 ± 4.5	0.974
WHR	0.92 ± 0.05	0.92 ± 0.05	0.792
Cholesterol [mg/dL]	207 ± 39	234 ± 44	<0.01
HDL-C [mg/dL]	55 ± 9	55 ± 12	0.921
LDL-C [mg/dL]	123 ± 36	147 ± 39	<0.01
TG [mg/dL]	144 ± 74	162 ± 74	0.295
Glucose [mg/dL]	97 ± 14	96 ± 12	0.615
TNFα [pg/mL]	8.21 ± 3.60	11.27 ± 7.30	0.117
IL6 [pg/mL]	2.20 ± 1.56	3.03 ± 3.49	0.216

Results are shown as group means with their standard deviations. BMI: body mass index; WHR: waist-to-hip ratio; HDL-C: high-density lipoprotein cholesterol; LDL-C: low-density lipoprotein cholesterol; TG: triglycerides; TNFα: tumor necrosis factor alpha; IL6: interleukin 6; The regression model for all the biochemical parameters was adjusted for BMI, all the parameters were adjusted for physical activity level.
